# Association between *ANRIL* polymorphisms and risk of obsessive-compulsive disorder

**DOI:** 10.1016/j.heliyon.2023.e14081

**Published:** 2023-02-25

**Authors:** Mohammadarian Akbari, Bashdar Mahmud Hussen, Solat Eslami, Seyedeh Morvarid Neishabouri, Soudeh Ghafouri-Fard

**Affiliations:** a Skull Base Research Center, Loghman Hakim Hospital, Shahid Beheshti University of Medical Sciences, Tehran, Iran; b Department of Clinical Analysis, College of Pharmacy, Hawler Medical University, Kurdistan Region, Erbil, Iraq; c Dietary Supplements and Probiotic Research Center, Alborz University of Medical Sciences, Karaj, Iran; d Department of Medical Biotechnology, School of Medicine, Alborz University of Medical Sciences, Karaj, Iran; e Department of Psychiatric, School of Medicine, Shahid Beheshti University of Medical Sciences, Tehran, Iran; f Department of Medical Genetics, Loghman Hakim Hospital, Shahid Beheshti University of Medical Sciences, Tehran, Iran

**Keywords:** ANRIL, lncRNA, Obsessive-compulsive disorder, rs1333045, rs1333048, rs10757278, rs4977574

## Abstract

Obsessive-compulsive disorder (OCD) is a disorder in which genetic factors participate. ANRIL is an example of long non-coding RNAs with crucial roles in the pathoetiology of multifactorial disorders, including neuropsychiatric conditions. We appraised association between rs1333045, rs1333048, rs10757278 and rs4977574 polymorphisms and OCD in Iranian population. There were no remarkable differences in allele and genotype distribution of rs1333045, rs1333048, rs4977574, and rs10757278 between OCD Patients and normal controls. However, the CCGG haplotype (equivalent to rs1333045, rs1333048, rs4977574 and rs10757278, respectively) has been shown to decrease risk of OCD (OR (95% CI) = 0.57 (0.39–0.85), P value-0.006 and FDR q-value = 0.041). On the other hand, TCGA haplotype has been found as a risk haplotype for OCD (OR (95% CI) = 5.17 (1.44–18.55), P value = 0.005 and FDR q-value = 0.041). In brief, the current study indicates association between two ANRIL haplotypes and risk of OCD in Iranian people.

## 1 Introduction

Obsessive-compulsive disorder (OCD) is described by disturbing unwanted thoughts and/or images and/or ritualized repetitive conducts (compulsions) [1]. OCD has a lifetime prevalence ranging from 1% to 3% in different regions [2,3], thus it is among prevalent psychiatric disorders. Based on twin and family studies, one can suggest important roles of genetic factors in the pathoetiology of OCD [4]. Moreover, candidate gene studies have reported associations between OCD and a number of genes within serotonergic, dopaminergic and glutamatergic systems [5]. Many studies have pointed to importance of long non-coding RNAs (lncRNAs) in multifactorial disorders [6]. Notably, *ANRIL* is an example of these transcripts with crucial roles in the pathoetiology of multifactorial disorders, including neuropsychiatric conditions [7], cancers and metabolic disorders [8]. Besides, *ANRIL* locus has been identified as a hot spot for human disorders, based on the results of genome wide association studies [9]. The rs1333045 and rs1333048 polymorphisms within *ANRIL* gene have been found to be associated with addiction to methamphetamine, major depressive disorder and bipolar disorder [7]. Yet, their association with OCD has not been assessed. Previous studies have suggested etiological overlaps between OCD and depressive symptoms. Notably, this overlap is due to common genetic factors rather than environmental factors [10]. Besides, the TAAA haplotype within this lncRNA (corresponding to rs1333045, rs1333048, rs4977574, and rs10757278 variants, respectively) has a tendency to be more common among autism spectrum disorder (ASD) children compared with normal children. In addition, the TAGG haplotype has an opposite trend [11]. It is worth mentioning that integrative genetic analyses have revealed significant overlap between genes associated with OCD and ASD. In fact, OCD and ASD might affect each other through a genetic network [12]. Another study in Iranian population has shown protective impact of the TAAA haplotype of *ANRIL* SNPs against development of multiple sclerosis (MS) [13].

Therefore, we planned the present case-control study to judge association between rs1333045, rs1333048, rs10757278 and rs4977574 polymorphisms and OCD in Iranian population.

## 2 Materials and methods

### 2.1 Patients and controls

This project was conducted on 120 OCD patients (87 women and 33 men, mean age ± SD: 37.85 ± 10.02) and 149 controls (96 females and 53 males, mean age ± SD: 36.58 ± 11.33). Cases were diagnosed based on DSM-5 criteria (2013). Controls were precisely assessed to exclude the presence of any psychiatric disorder. Moreover, they did not have positive family history for psychiatric disorders. Written informed consent was obtained from participants. The study protocol was confirmed by ethical committee of Shahid Beheshti University of Medical Sciences (IR.SBMU.MSP.REC.1400.301).

### 2.2 Genotyping

The rs1333045, rs1333048, rs10757278 and rs4977574 SNPs of *ANRIL* were genotyped using T-primer ARMS-PCR method using primers and methods explained formerly [7,14]. PCR program included an initial denaturing step at 95 °C for 10 min; 35 cycles at 94 °C for 30 s, 52 or 53 °C for 30 s, 72 °C for 60 s, and an ultimate extension at 72 °C for 7 min. Genotyping methods were performed in the FlexCycler system (Analytik Jena, Germany) using Taq 2× red master mix (Ampliqon, Denmark).

### 2.3 Statistical analysis

SPSS v.22.0 and SNP Analyzer 2.0 were used for statistical analyses. Alleles and genotypes frequencies were compared between OCD cases and controls using the chi-squared test. Odds ratios (ORs) for effect alleles and genotypes were measured by logistic regression. Adjusted relative risks were measured using sex as a covariate. Association between genomic variants and OCD risk was assessed in four inheritance models. Associations were described as OR and 95% confidence interval of OR (95% CI), *P*-value and FDR adjusted q-values. The FDR adjusted q-values were estimated via analyzing a stack of p values in column analyses by GraphPad Prism version 9.0. P value < 0.05 was considered as significant.

Association of haplotypes with OCD was investigated using a haplotype-specific test with one degree-of-freedom. D′ and r parameters were calculated for assessment of linkage between rs1333045, rs1333048, rs10757278 and rs4977574 variants.

## 3 Results

**Fig. 1:**
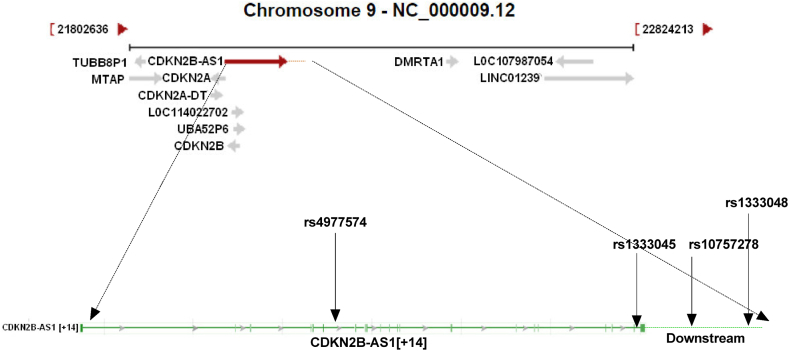
Locations of rs1333045, rs1333048, rs10757278 and rs4977574 variants in the ANRIL gene and its downstream. The rs4977574 and rs1333045 are located in the introns, at positions 22098575 and 22119196, respectively. The rs10757278 and rs1333048 are located in the downstream region of the *ANRIL* gene at positions 22124478 and 22125348, respectively.

Fig. 1 shows the location of selected SNPs in *ANRIL*. As depicted in the figure, the rs4977574 and rs1333045 are located in the introns, while the rs10757278 and rs1333048 are located in the downstream region of the *ANRIL* gene.

Based on the SNPedia, the minor/major alleles of rs1333045, rs1333048, rs4977574 and rs10757278 are C/T, C/A, A/G and A/G, respectively. Table 1 shows the detailed information about these SNPs.

**Table 1:** Descriptive information of four SNPs tested for association with OCD patients.

Gene	SNP ID	Position	Functions and SNP Types	Minor/Major allele
CDKN2B-AS1	rs1333045	Chr9:22119196	Intron, Transition Substitution	C/T
CDKN2B-AS1	rs1333048	Chr9:22125348	Downstream, Transition Substitution	C/A
CDKN2B-AS1	rs4977574	Chr9:22098575	Intron, Transition Substitution	A/G
CDKN2B-AS1	rs10757278	Chr9:22124478	Downstream, Transition Substitution	A/G

**Fig. 2:**
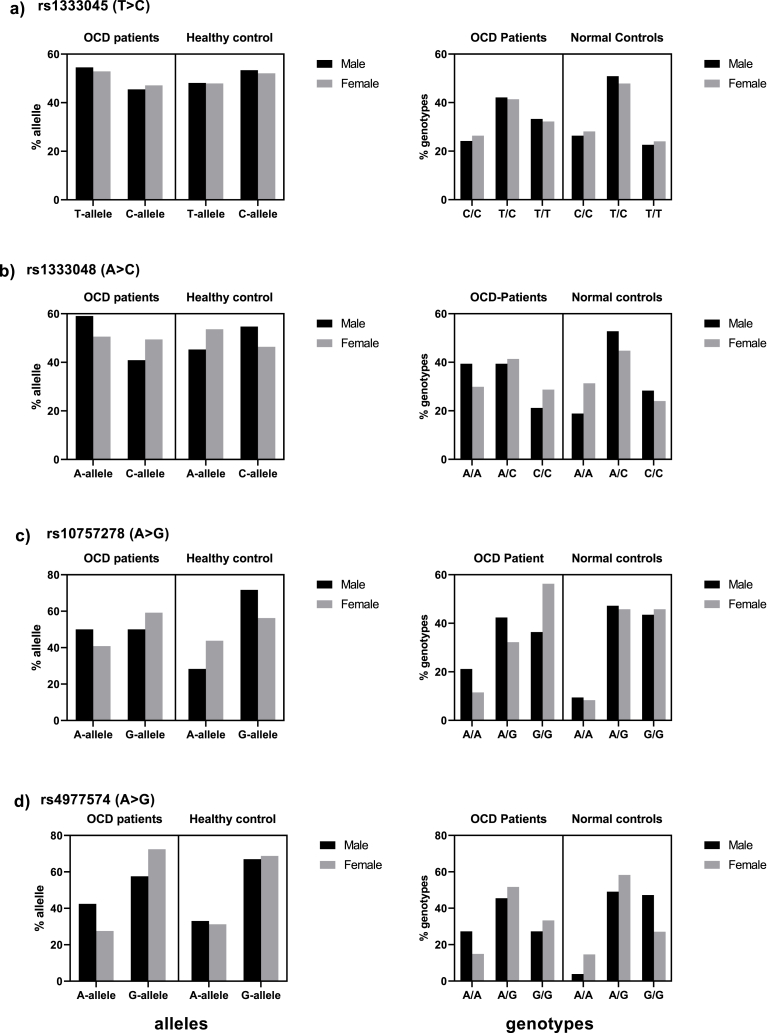
Allele and genotype distribution of rs1333045, rs1333048, rs10757278 and rs4977574 between OCD patients and normal controls and subgroups at gender level.

Analyses using chi-square test of association and the obtained P values showed no significant difference in allele and genotype distribution of rs1333045, rs1333048, rs4977574, and rs10757278 between OCD patients and normal controls. P values for association analyses of genotypes were 0.24, 0.48, 0.11 and 0.2 for rs1333045, rs1333048, rs4977574 and rs10757278, respectively. The corresponding P values for association analyses of alleles of these SNPs were 0.24, 0.73, 0.89 and 0.23, respectively. Alleles and genotypes distribution are shown in Table 2 and Fig. 2.

**Table 2:** Genotypes and allele frequencies of the four SNPs of *ANRIL* gene in OCD patients and normal controls.

SNPs	Gender	N	Genotype				Allele		
			1/1	1/2	2/2	χ2 P	1	2	χ2 P
rs1333045 (T > C)	OCD	120	39 (32.5)	50 (41.7)	31 (25.8)	2.81 0.24	128 (53.3)	112 (46.7)	1.33 0.24
	NC	149	35 (23.5)	73 (49)	41 (27.5)		144 (48)	154 (52)	
rs1333048 (A > C)	OCD	120	39 (32.5)	49 (40.8)	32 (26.7)	1.45 0.48	126 (52.9)	114 (47.1)	0.11 0.73
	NC	149	40 (26.8)	71 (47.7)	38 (25.5)		152 (50.7)	146 (49.3)	
rs4977574 (A > G)	OCD	120	17 (14.2)	42 (35)	61 (50.8)	4.3 0.11	76 (31.7)	164 (68.3)	0.01 0.89
	NC	149	13 (8.7)	69 (46.3)	67 (45)		96 (31.9)	202 (68.1)	
rs10757278 (A > G)	OCD	120	22 (18.3)	60 (50)	38 (31.7)	3.16 0.2	104 (43.3)	136 (56.7)	1.42 0.23
	NC	149	16 (10.7)	82 (55)	51 (34.2)		114 (38.3)	184 (61.7)	

1/1 homozygous reference; 1/2, heterozygous; 2/2, homozygous mutant; 1, wild allele; 2, mutant allele (based on SNP database); OCD, Obsessive-compulsive disorder; NC, normal control.

According to the SNP database, the wildtype allele for rs1333045 is T. For rs1333048, rs4977574 and rs10757278, the wildtype allele is A allele. The allele C (for rs1333045 and rs1333048), and A (for rs4977574 and rs10757278) were the minor alleles in this study and considered as effect alleles.

Table 3 demonstrates the results of evaluation of accordance with Hardy-Weinberg equilibrium. Distribution of rs1333048 genotypes in OCD patients and distribution of rs10757278 alleles in controls were not in accordance with this equilibrium (P value = 0.04 for both comparisons).

**Table 3:** Results of assessment of accordance of *ANRIL* SNPs with HWE.

Groups	rs1333045				rs1333048				rs4977574				rs10757278			
	CC	CT	TT	P value	AA	A C	CC	P value	AA	AG	GG	P value	AA	AG	GG	P value
OCD patients	31	50	39	0.07	39	49	32	0.04	17	42	61	0.036	22	60	38	0.84
Normal controls	41	73	35	0.82	40	71	38	0.56	13	69	67	0.41	16	82	51	0.04

After adjustment for the effects of gender, there was no significant association between *ANRIL* polymorphisms and risk of OCD in co-dominant, dominant, recessive or over-dominant models (Table 4).

**Table 4:** Association between *ANRIL* genotypes and risk of OCD.

rs ID	Models	Genotypes	Case number (%)	Control number (%)	OR (95% CI) (1)	p-Value (1)	FDR q-Value (1)	OR (95% CI) (2)	p-Value (2)	FDR q-Value (2)
rs1333045	Co-dominant	CC vs. TT	31 (25.8)	41 (27.5)	1.1 (0.61–1.99)	0.74	0.79	1.09 (0.6–1.97)	0.76	0.80
		TC vs. TT	50 (41.7)	73 (49)	0.9 (0.5–1.63)	0.74	0.79	0.91 (0.5–1.65)	0.76	0.80
	Dominant	CC + TC vs. TT	81 (67.5)	114 (76.5)	1.56 (0.91–2.68)	0.1	0.48	1.56 (0.91–2.68)	0.1	0.50
	Recessive	CC vs. TC + TT	31 (25.8)	41 (27.5)	1.09 (0.63–1.87)	0.75	0.79	1.09 (0.63–1.89)	0.73	0.80
	Over dominant	CC + TT vs. TC	70 (58.3)	76 (51)	0.74 (0.45–1.2)	0.23	0.48	0.74 (0.46–1.21)	0.24	0.50
rs1333048	Co-dominant	CC vs. AA	32 (26.7)	38 (25.5)	1.22 (0.67–2.21)	0.51	0.76	1.21 (0.66–2.19)	0.53	0.78
		AC vs. AA	49 (40.8)	71 (47.7)	0.82 (0.45–1.48)	0.51	0.76	0.82 (0.45–1.5)	0.53	0.78
	Dominant	CC + AC vs. AA	81 (67.5)	109 (73.2)	1.31 (0.77–2.22)	0.31	0.76	1.29 (0.76–2.19)	0.33	0.78
	Recessive	CC vs. AC + AA	32 (26.7)	38 (25.5)	0.94 (0.54–1.62)	0.82	0.86	0.94 (0.54–1.63)	0.83	0.87
	Over dominant	CC + AA vs. AC	71 (59.2)	78 (52.3)	0.75 (0.46–1.23)	0.26	0.76	0.76 (0.47–1.25)	0.28	0.78
rs4977574	Co-dominant	AA vs. GG	17 (14.2)	13 (8.7)	2.14 (0.94–4.86)	0.067	0.141	2.2 (0.96–5.01)	0.06	0.147
		AG vs. GG	42 (35)	69 (46.3)	0.46 (0.2–1.05)	0.067	0.141	0.45 (0.2–1.03)	0.06	0.147
	Dominant	AA + AG vs. GG	59 (49.2)	82 (55)	1.26 (0.78–2.04)	0.33	0.416	1.22 (0.75–1.99)	0.4	0.504
	Recessive	AA vs. AG + GG	17 (14.2)	13 (8.7)	0.57 (0.26–1.24)	0.16	0.252	0.55 (0.25–1.2)	0.13	0.205
	Over dominant	AA + GG vs. AG	78 (65)	80 (53.7)	0.62 (0.38–1.02)	0.06	0.141	0.63 (0.38–1.04)	0.07	0.147
rs10757278	Co-dominant	AA vs. GG	22 (18.3)	16 (10.7)	1.87 (0.91–3.88)	0.08	0.185	1.88 (0.91–3.9)	0.087	0.183
		AG vs. GG	60 (50)	82 (55)	0.53 (0.25–1.09)	0.088	0.185	0.53 (0.25–9)	0.087	0.183
	Dominant	AA + AG vs. GG	82 (68.3)	98 (65.8)	0.89 (0.53–1.48)	0.65	0.683	0.92 (0.54–1.54)	0.75	0.788
	Recessive	AA vs. AG + GG	22 (18.3)	16 (10.7)	0.53 (0.26–1.07)	0.078	0.185	0.54 (0.26–1.08)	0.08	0.183
	Over dominant	AA + GG vs. AG	60 (50)	67 (45)	0.81 (0.5–1.32)	0.41	0.517	0.79 (0.48–1.29)	0.35	0.441

(1) Unadjusted, (2) adjusted by sex. OR: Odds ratio; FDR: false discovery rate.

OCD-associated haplotypes were different from ASD-associated and MS-associated haplotypes in Iranian population. The CCGG haplotype (corresponding to rs1333045, rs1333048, rs4977574 and rs10757278, respectively) has been shown to decrease risk of OCD (OR (95% CI) = 0.57 (0.39–0.85), P value-0.006 and FDR q-value = 0.041). On the other hand, TCGA haplotype has been found as a risk haplotype for OCD (OR (95% CI) = 5.17 (1.44–18.55), P value = 0.005 and FDR q-value = 0.041) (Table 5).

**Table 5:** Haplotype analysis of *ANRIL* in OCD patients and control group.

rs1333045	rs1333048	rs4977574	rs10757278	Freq. in Case	Freq. in Control	Total Freq.	OR (95%CI)	*P*-value	FDR q-Value
**C**	**C**	**G**	**G**	0.15	0.27	0.21	**0.57 (0.39**–**0.85)**	**0.006**	0.041*
T	A	G	A	0.11	0.11	0.11	1.03 (0.61–1.74)	0.9	0.874
T	C	G	G	0.10	0.093	0.10	1.39 (0.76–2.5)	0.27	0.614
T	A	A	A	0.077	0.11	0.09	0.91 (0.55–1.49)	0.71	0.808
C	A	G	G	0.10	0.047	0.07	1.94 (0.96–3.91)	0.058	0.264
T	A	G	G	0.046	0.056	0.053	0.79 (0.36–1.72)	0.56	0.695
C	A	A	G	0.069	0.034	0.05	1.9 (0.76–4.74)	0.15	0.410
C	A	G	A	0.037	0.052	0.048	0.92 (0.31–2.71)	0.89	0.874
C	A	A	A	0.045	0.049	0.047	0.76 (0.34–1.72)	0.51	0.695
T	C	A	G	0.033	0.047	0.041	0.68 (0.22–2.06)	0.49	0.695
**T**	**C**	**G**	**A**	0.076	0.009	0.04	**5.17 (1.44**–**18.55)**	**0.005**	0.041*
T	A	A	G	0.032	0.034	0.032	0.73 (0.26–2.06)	0.56	0.695
C	C	G	A	0.037	0.025	0.03	1.76 (0.55–5.61)	0.33	0.644
C	C	A	G	0.014	0.023	0.023	0.74 (0.17–3.13)	0.96	0.874
T	C	A	A	0.043	0.006	0.022	3.39 (0.89–12.92)	0.11	0.375
C	C	A	A	–	0.0078	0.004	–		

**Fig. 3:**
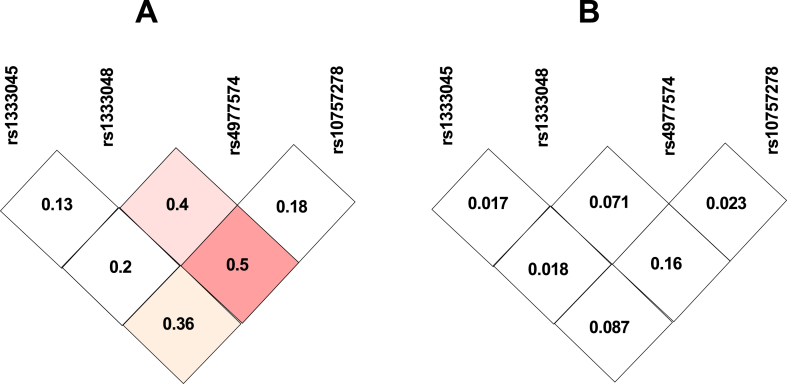
Linkage disequilibrium plot of four SNPs in the *ANRIL* gene. (A) D′ value, (B)r2 value.

Finally, we assessed linkage disequilibrium between mentioned SNPs (Table 6 and Fig. 3). Based on D′ and r values obtained from SNP Analyzer 2.0, these SNPs were not in linkage disequilibrium.

**Table 6:** D’ and r parameters for estimation of linkage disequilibrium between SNPs.

SNPs	D’ statistic				r statistic			
	rs1333045	rs1333048	rs4977574	rs10757278	rs1333045	rs1333048	rs4977574	rs10757278
rs1333045	.	0.13	0.40	0.18	.	0.017	0.071	0.023
rs1333048	.	.	0.2	0.5	.	.	0.018	0.16
rs4977574	.	.		0.36	.	.	.	0.087

## 4 Discussion

OCD is a multifactorial disorder resulting from a combination of genetic and environmental elements [15]. Family and twin studies have demonstrated impact of genetic factors in the pathetiology of OCD [15].

Genome wide studies have shown association between a number of loci and OCD. For instance, significant association has been found for a marker on chromosome 9, adjacent to *PTPRD* gene, a gene that regulates differentiation of glutamatergic synapses [16]. *DLGAP1*, a member of the neuronal postsynaptic density complex has been identified to be a risk locus in another GWAS [17].

Recent studies have shown that lncRNA might have pathogenic implications in neuropsychiatric disorders, contributing in the onset and progression of these disorders. Moreover, a group of lncRNAs have been found to exert homeostatic effects in these contexts. Examples of this class of lncRNAs are those whose variations represent an attempt to defeat pathophysiology of these disorders [18]. *ANRIL* is a lncRNA which participates in the pathoetiology of neuropsychiatric conditions [7]. The rs1333045 and rs1333048 polymorphisms within *ANRIL* gene have been found to be associated with addiction to methamphetamine, major depressive disorder and bipolar disorder [7]. Also, a recent study has shown that TAAA haplotype has a tendency to be more common among ASD children compared with. In addition, the TAGG haplotype has an opposite trend [11]. Another study in Iranian population has shown protective impact of the TAAA haplotype against development of MS [13]. Moreover, a study in Finish population has detected association between *ANRIL* and Hypomanic Personality Scale (HPS), HPS-Perceptual Aberration Scale, HPS-Revised Social Anhedonia Scale and HPS- Revised Physical Anhedonia Scale [19].

We genotyped four SNPs in this lncRNA in a population of OCD patients and controls. We detected no significant differences in alleles and genotypes distribution of rs1333045, rs1333048, rs4977574, and rs10757278 between OCD patients and normal controls. However, the CCGG haplotype has been shown to decrease risk of OCD. On the other hand, TCGA haplotype has been found as a risk haplotype for OCD. This finding indicates possible role of *ANRIL* in conferring risk of OCD among Iranians. Notably, OCD-associated haplotypes have been different from ASD-associated and MS-associated haplotypes in this population, indicating distinctive roles of this lncRNA in different neuropsychiatric conditions.

Mechanistically, *ANRIL* can serve as a sponge for some miRNAs, therefore affecting the expression of mRNA targets for these miRNAs. It is possible that a number of these miRNAs affect the activity of main signaling pathways that are responsible for induction of OCD phenotype. Therefore, the distinctive distribution of *ANRIL* haplotypes between OCD patients and controls can be explained by the effects of these haplotypes on the activity and functions of *ANRIL* in the regulation of these pathways.

In a few words, the current study indicates association between two *ANRIL* haplotypes and risk of OCD in Iranian population. Further association studies along with expression analyses in large cohorts of OCD patients are required for appraisal of contribution of *ANRIL* variants in OCD pathogenesis. Our study has limitations in terms of small sample size and lack of functional studies to unravel the mechanism of contribution of mentioned SNPs in the risk of OCD.

## Declarations

### Ethics approval and consent to participant

All procedures were in accordance with the ethical standards of national research committee and with the 1964 Helsinki declaration. Informed consent forms were obtained from all study participants. The study protocol was approved by the ethical committee of Shahid Beheshti University of Medical Sciences.

## Author contribution statement

Mohammadarian Akbari, Seyedeh Morvarid Neishabouri: Performed the experiments; Analyzed and interpreted the data; Contributed reagents, materials, analysis tools or data.

Bashdar Mahmud Hussen: Conceived and designed the experiments; Performed the experiments; Analyzed and interpreted the data; Contributed reagents, materials, analysis tools or data; Wrote the paper.

Solat Eslami: Analyzed and interpreted the data; Contributed reagents, materials, analysis tools or data.

Soudeh Ghafouri-Fard: Conceived and designed the experiments; Wrote the paper.

## Data availability statement

The datasets used and/or analyzed during the current study are available from the corresponding author on reasonable request.

## Declaration of interest's statement

The authors declare no conflict of interest.
